# 非小细胞肺癌患者肿瘤组织与外周血中EGFR表达相关性研究

**DOI:** 10.3779/j.issn.1009-3419.2010.12.05

**Published:** 2010-12-20

**Authors:** 琛 郭, 其森 郭, 洪生 曾, 银玲 禚, 燕 管, 秀菊 刘

**Affiliations:** 1 256603 滨州，滨州医学院附属医院肿瘤内科 Department of Medical Oncology, Afliated Hospital of Binzhou Medical College, Binzhou 256603, China; 2 250117 济南，山东省肿瘤医院内科 Department of Internal Medicine, Shandong Tumor Hospital, Jinan 250117, China

**Keywords:** 肺肿瘤, EGFR, 血浆, 肿瘤组织, RT-PCR, Lung neoplasms, EGFR, Plasma, Tumor tissue, RT-PCR

## Abstract

**背景与目的:**

表皮生长因子受体（epidermal growth factor receptor, EGFR）在肿瘤细胞的增殖中起重要作用，肿瘤组织中EGFR表达水平可为肺癌的诊断、预后判断提供依据。本研究通过检测EGFR在非小细胞肺癌患者肿瘤组织和外周血中的表达，探讨利用外周血中EGFR表达水平来预测肿瘤组织中的表达并作为肿瘤标记物的可行性。

**方法:**

应用real-time RT-PCR技术检测46例非小细胞肺癌组织和10例肺良性病变组织中EGFR mRNA的表达；应用ELISA法检测46例非小细胞肺癌患者外周血中EGFR蛋白的表达，并以10例肺良性疾病患者的外周血作对照。

**结果:**

直线相关分析表明EGFR在外周血中的蛋白表达水平与肿瘤组织中的mRNA表达水平具有相关性（*R*^2^=0.83, *P*=0.016）。

**结论:**

血清中EGFR蛋白水平的测定有可能替代肿瘤组织中mRNA的检测，为无法取得肿瘤组织或取到肿瘤组织困难的患者提供一种更快捷、简便的替代检测方法，尽早为临床选择治疗方法及预后判断提供依据。

## Abstract (en)

**Background and objective:**

It has been proven that EGFR plays an important role in cell proliferation, and has predictive values for diagnosis and prognosis. The aim of this study is to investigate the correlation between the expression of EGFR in tumor tissue and peripheral blood in non-small cell lung cancer (NSCLC) patients.

**Methods:**

The expression of EGFR was analyzed by real-time RT-PCR in 46 NSCLC tumor tissues and by ELISA in peripheral blood, compared with 10 patients with benign pulmonary diseases as control.

**Results:**

Linear correlation analysis revealed there was a correlation of the expression of EGFR protein between peripheral blood and tumor tissue (*P*=0.016, *R*^2^=0.83).

**Conclusion:**

The expression of EGFR mRNA in tumor tissue was positively correlated with EGFR protein in plasma. Detection of protein in plasma may replace that in tumor tissue, providing a quicker and easier examination method for the patients lacking biopsy tissue.

表皮生长因子受体（epidermal growth factor receptor, EGFR）是一种细胞膜表面的糖蛋白受体，具有酪氨酸激酶活性，是原癌基因*C-erb-1*的表达产物。EGFR的高表达可以促进肿瘤血管生成以及肿瘤细胞的增殖、粘附、侵袭、转移^[1]^。正常肺组织中，EGFR低表达或不表达，而在非小细胞肺癌（non-small cell lung cancer, NSCLC）中EGFR常表现为活性增高^[2]^，据文献报道其表达率为23%-89%。

尽管组织学类型、临床分期相同且采用的治疗方法相同，部分患者却出现不同的结果。因此，利用已知肿瘤细胞特点作为个体化治疗的探索，有助于提高疗效。本研究旨在通过对分子学指标的检测为治疗方式的选择、术后的辅助治疗、预测化疗耐药、提供阻断方法、评价预后提供更准确的依据。

## 1 材料和方法

### 1.1 一般资料

选取2007年1月-2007年10月在山东省肿瘤医院胸外科行手术切除的NSCLC患者46例为病例组及良性肺病变患者10例做对照组。病例组男性38例，女性8例；年龄36岁-74岁，中位年龄61.5岁。不吸烟者18例，≤400年支者7例， > 400年支者21例。腺癌21例，鳞癌20例，其它5例。Ⅰ期11例，Ⅱ期14例，Ⅲa期19例，Ⅳ期2例。

### 1.2 试剂与引物设计

羊抗人EGFR ELISA试剂盒为美国Adlitteram Diagnostic Laboratories产品。采用QIAGEN公司的AllPrep DNA/RNA Mini Kit试剂盒抽提组织标本中的RNA，反转录试剂盒Script^TM^ RT reagent kit（Perfect Real Time）为TaKaRa公司产品。PCR试剂包括：dNTP（10 mM）（GENERAY BIOTECH公司）、PCR Buffer（10 ×）（QIAGEN公司）、Hotstar taq（QIAGEN公司）、PCR产物纯化试剂盒（CR Purification Kit: To Pure^TM^, Gene Tech）、real-time PCR试剂盒[SYBR Premix Ex Taq^TM^（Perfect Real Time），TaKaRa公司]。采用Primer Premier5软件设计引物，EGFR上游：5'-GGACTCTGGATCCCAGAAGGT G-3'，EGFR下游：5'-GCTGGCCATCACGTAGGCTT-3'，产物大小为244 bp；内参引物为GAPDH，GAPDH上游：5'-CAACAGCCTCAAGATCATCAGC-3'，GAPDH下游：5'- TTCTAGACGGCAGGTCAGGTC-3'，产物大小为306 bp；由上海生工生物工程技术服务有限公司合成。

### 1.3 标本的收集

病例组采集术后新鲜肿瘤组织，对照组采集病灶组织。每例均采集正常肺组织及手术前6 h、手术后24 h外周静脉血4 mL。组织标本及分离出的血浆置入-80 ℃冰箱中保存。

### 1.4 EGFR在肿瘤组织中表达的检测

RNA抽提完成后，将RNA反转录为cDNA，反应条件为37 ℃、15 min，85 ℃、5 s。以cDNA为模板进行PCR扩增目的片段EGFR和GAPDH，PCR反应条件为95 ℃、15 min，94 ℃、30 s，63 ℃、1 min（touch down，每个循环减0.5 ℃），72 ℃、1 min（15个循环），94 ℃、30 s，56 ℃、1 min，72 ℃、1 min（30个循环），72 ℃、10 min。PCR产物需电泳鉴定及纯化，并用标准品DNA对PCR产物进行定量。完成后梯度稀释制作标准曲线进行real-time PCR，反应条件为95 ℃、10 s，95 ℃、5 s，57 ℃、20 s，72 ℃、30 s（40个循环），95 ℃、15 s，60 ℃、1 min，95 ℃、15 s（即溶解曲线）。采用绝对定量的方法进行real-time RT-PCR的检测，根据样品的CT值可以从标准曲线上读出各个样品的浓度（拷贝数/mL），计算出目的基因的拷贝数与相同样本的管家基因的拷贝数的比值作为目的基因的表达水平，从而得到计量资料（图 1）。

**1:**
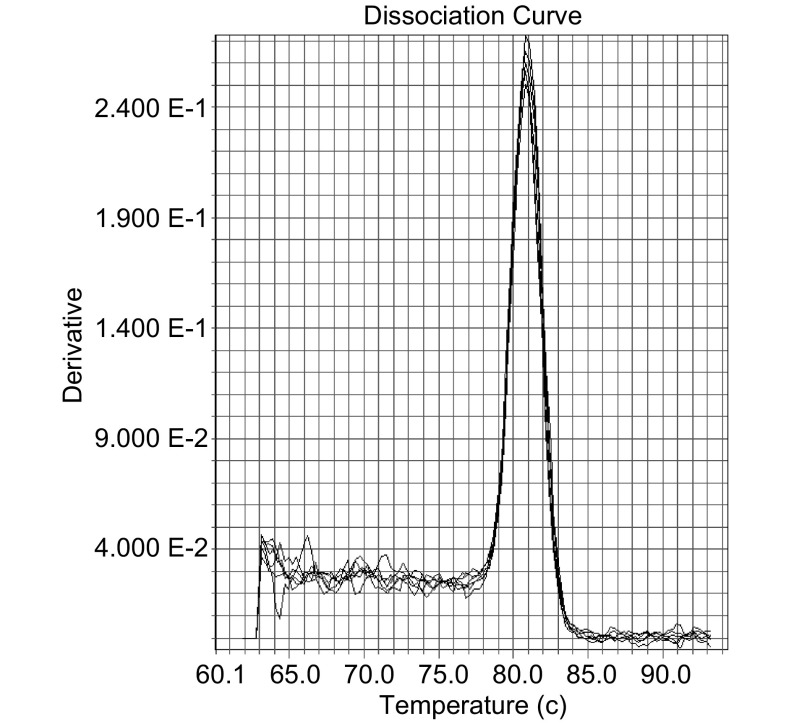
Real-time RT-PCR结果 Result of real-time RT-PCR

### 1.5 EGFR在外周血中表达的检测

按试剂盒说明对血浆标本进行ELISA检测，在全波长扫描酶标仪450 nm处检测光密度（optical density, OD）值。EGFR标准品的浓度分别为0、50 pg/mL、100 pg/mL、200 pg/mL、400 pg/mL、800 pg/mL，试剂盒的最大检测浓度为800 pg/mL，检测灵敏度为1.0 pg/mL。标准曲线方程为Y=144.93X-14，*R*^2^=0.997 7。

### 1.6 统计学方法

应用SPSS 10.0软件进行统计分析，检验水准*α*=0.05（双侧）。所有计量资料均应先经过单样本*K-S*检验，判断是否为正态分布。符合双样本均为正态分布的计量资料比较采用独立样本*t*检验、配对*t*检验、方差分析、直线相关分析等方法，*P* < 0.05为差异有统计学意义。

## 2 结果

### 2.1 EGFR在外周血中的表达

#### 2.1.1 与临床病理特征的关系

血浆EGFR蛋白表达水平与性别、年龄、肿瘤大小、病理类型、肿瘤分化程度等因素无关，与有无淋巴结转移、吸烟指数有关（表 1）。

**1:** EGFR手术前外周血中表达水平与临床病理特征的相关性 Relationship of the EGFR expression in peripheral and blood clinicopathological characteristics between groups before operation

Factor	*n*	Concentration (pg/mL)	*t*	*P*
Age (years)			-0.232	0.818
< 60	18	4 705±2 310		
≥ 60	28	5 303±4 184		
Gender			0.642	0.524
Male	38	5 262±3 829		
Female	8	4156±1 549		
Histological type			0.393	0.678
Adenocarcinoma	21	5 872 ±4 907		
Squamous cell carcinoma	20	4 324±1 704		
Others	5	4 675±1 147		
Differentiation			0.588	0.561
High	16	8 660±9 621		
Medium	23	4 258±2 427		
Low	7	4 326±1 950		
Tumor size (diameter)			-0.619	0.539
≤3 cm	11	4 304±1 717		
> 3 cm	35	5 309±3 946		
Metastases			1.86	0.076
Yes	31	4 625±2 559		
No	15	4 524±2 061		
Stage			0.191	0.902
Ⅰ	11	6 993±6 576		
Ⅱ	14	4 921±2 861		
Ⅲ	19	5 251±2 725		
Ⅳ	2	4 434±1 439		
Smoking Index			-2.048	0.047
≤ 400	25	3 913±1 753		
> 400	21	4 936±1 752		

#### 2.1.2 肿瘤与良性疾病的比较

NSCLC患者中EGFR表达为（5 069±3 550）pg/mL，良性肺部疾病患者中EGFR表达为（4 930±2 097）pg/mL，两者相比差异无统计学意义（*t*=0.086, *P*=0.932）。

#### 2.1.3 手术前后的比较

NSCLC患者术前EGFR表达为（4 777±2 150）pg/mL，术后为（2 960±1 006）pg/mL，两者比较差异具有统计学意义（*t*=5.28, *P* < 0.001）。在手术后患者的表达水平上，腺癌组为（3 703±1 138）pg/mL，鳞癌组为（2 655±804）pg/mL，腺鳞癌组为（2 374± 533）pg/mL，腺癌组与鳞癌组间存在明显差异（*t*=2.96, *P*=0.006），腺癌组与腺鳞癌组间亦有明显差异（*t*=2.22, *P*=0.04）。

### 2.2 EGFR在肿瘤组织中的表达

肿瘤组织中EGFR mRNA的表达与性别、年龄、肿瘤大小、病理类型、肿瘤分化程度、吸烟等因素无关，与有无淋巴结转移有关（表 2）。

**2:** EGFR在癌组织中的表达与临床病理特点的相关性 Correlation of the EGFR expression in tumor tissue and clinicopathological chara cteristics between groups after operation

Variable	*n*	Expression	*t*	*P*
Age (years)			1.125	0.178
< 60	18	5.16±1.69		
≥60	28	5.83±1.74		
Gender			0.024	0.963
Male	38	5.68±1.96		
Female	8	5.54±1.88		
Histological type			-0.609	0.492
Adenocarcinoma	21	5.69±1.66		
Squamous cell carcinoma	20	5.84±2.07		
Others	5	5.45±1.82		
Differentiation			1.285	0.174
High	16	5.19±1.87		
Medium	23	5.81±2.01		
Low	7	5.45±1.34		
Tumor size (diameter)			1.084	0.207
≤3 cm	11	5.32±1.39		
> 3 cm	35	5.79±1.97		
Metastases			2.22	0.04
Yes	31	6.01±2.03		
No	15	4.65±1.96		
Stage			0.602	0.557
Ⅰ	11	5.88±1.21		
Ⅱ	14	5.46±1.48		
Ⅲ	19	5.80±1.73		
Ⅳ	2	5.26±0.89		
Smoking index			1.124	0.202
≤400	25	5.44±1.36		
> 400	21	5.75±1.29		

### 2.3 EGFR在肿瘤组织中与外周血中表达的相关性分析

采用直线相关的方法对肿瘤组织和外周血中EGFR的表达进行相关性的分析。利用同一样本的GAPDH作为EGFR的内参进行组织样本的检测。血浆中EGFR的表达水平与NSCLC肿瘤组织中EGFR mRNA的表达水平呈正相关（*R*^2^=0.83, *P*=0.016）（图 2）。肿瘤组织中EGFR的表达与外周血中的表达均显示出明显的相关性，肿瘤组织中表达水平越高，外周血中的蛋白表达水平越高。这些数据提示：外周血中的蛋白可能来源于肿瘤组织的分泌。

**2:**
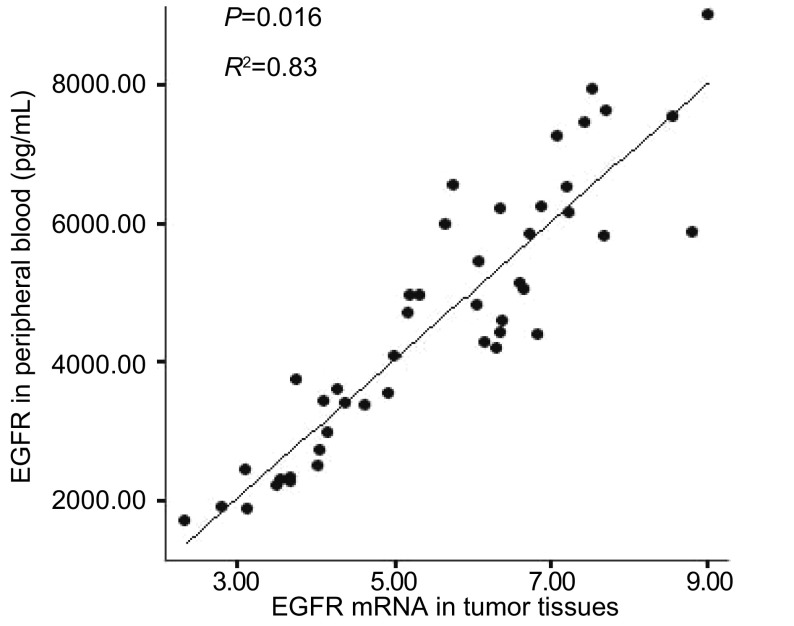
EGFR在肿瘤组织与外周血中表达的相关性 Correlation between the EGFR expression in tumor tissue and in peripheral blood

## 3 讨论

肺癌已成为我国第一大癌症，且发病率和死亡率增长最为迅速。淋巴结转移是NSCLC的主要转移途径之一。胸部CT检查是最常用的NSCLC肿瘤分期和淋巴结转移情况评估的非创伤性检查，主要是通过淋巴结的大小和形状来判断是否有转移，但这样可能漏诊一些较小的转移淋巴结或者误诊较大的非转移性淋巴结（如炎症）。纵隔镜和PET可进行更精确的淋巴结转移检查。血液中相关因子的检测创伤小并且费用低，在没有PET的医院里或患者承受高额费用较困难时，是一种更可行的途径。分子生物学以肿瘤发生发展机制相关的肿瘤基因、肿瘤基因蛋白产物、生长因子及受体家族等为标记物，运用现代技术如免疫组化、PCR、流式细胞仪等，主要对原发瘤、淋巴结、骨髓、外周血高特异性高敏感性检测临床常规检查未发现的肿瘤散播，从而为肺癌早期诊断转移或微转移（micrometastasis）、治疗方式的选择、术后的辅助治疗、预测化疗耐药、提供阻断方法、评价预后提供更准确的依据。

EGFR信号通路主要分为：配体诱导胞外域构象变化、跨膜信号转导、胞内域形成激酶活性与下游信号激活、信号灭活四部分。EGF诱导EGFR胞外域进行构象变化，通过二聚化/寡聚化解除胞外域对胞内域激酶活性形成的抑制作用，促使胞内域相互作用形成激酶活性并完成胞内域酪氨酸残基的磷酸化。磷酸化的胞内域作为结合位点（docking sites）招募其它信号分子并依赖RTK活性完成对下游信号分子的磷酸化，启动信号转导通路。信号激活与灭活的协调控制是生物体正常机制实现的基础，EGFR信号灭活过程主要有内化与降解两部分^[3]^。

在NSCLC中，多项研究进行了血清EGFR蛋白表达的检测。Sasaki^[4]^的研究共有106例肺癌患者和16例良性病变患者入组，所有患者都在未进行任何治疗前采集外周血标本。肺癌组（21.275±22.035）fm/mL与良性对照组（22.630±7.330）fm/mL表达无明显差异（*P*=0.808 3），说明血清EGFR蛋白的表达在鉴别良恶性方面有一定的局限性。肺癌患者中有淋巴结转移组（23.515±20.065）fm/mL和无淋巴结转移组（16.390±10.970）fm/mL的表达水平有明显差异（*P*=0.022 8），说明血清EGFR蛋白的表达在判断淋巴结转移方面有一定的意义。肺癌的不同病理类型中表达无明显差异，血清EGFR正常组和血清EGFR升高组预后无明显差异。Miura^[5]^采用肺癌患者外周血抽提的mRNA进行real-time RT-PCR研究其作为肿瘤标记物的价值和意义，共112例肺癌患者和80例非恶性肿瘤患者入组，研究结果表明血清EGFR mRNA与肿瘤数目、临床分期有关（*P* < 0.05）。血清EGFR mRNA对肺癌诊断的敏感性和特异性分别是71.3%和80.0%。在肺癌组中，血清EGFR mRNA与肿瘤组织中EGFR mRNA呈明显的相关性（*P* < 0.05）。

本研究选择新鲜的冰冻癌组织进行EGFR研究，较石蜡包埋的组织，新鲜冰冻肿瘤组织是更好的研究对象，研究结果更可靠^[6]^。以往研究^[7]^中较多采用免疫组化来对肿瘤组织中的表达进行检测，另外还有荧光原位杂交、CISH^[8]^等技术。本研究采用real-time RT-PCR法进行研究，对表达水平进行定量，得到计量资料，使相关性的分析更加精确。实时荧光定量PCR技术是通过对PCR反应扩增中每一个循环产物荧光信号的实时检测从而实现对起始模板的定量和定性分析。在实时荧光定量PCR反应中，引入一种荧光化学物质，随着PCR反应的进行，PCR反应产物不断累积，荧光信号强度也等比例增加。每经过一个循环，收集一个荧光信号强度，这样就能通过荧光信号强度的变化监测产物量的变化。表示每个PCR反应管内荧光信号到达设定的阈值时所经历的循环数即为CT值。通过获得未知样品的CT值可以从标准曲线上读出样品的拷贝数。管家基因的表达相对恒定。

本研究表明：①经直线相关分析，EGFR在肿瘤组织中的表达与外周血中表达的水平存在相关性（*R*^2^=0.83, *P*=0.016）。外周血检测是一种创伤小而且更加方便可行的检测方法，外周血中EGFR与肿瘤组织的表达具有相似的生物学标记的意义；②肿瘤组织和外周血中EGFR高表达水平与淋巴结转移有关，可作为肺癌淋巴结转移的标记物；③NSCLC患者血清EGFR蛋白表达水平与性别、年龄、肿瘤大小、分化程度、病理类型无关；④外周血中EGFR的表达方面，吸烟指数≤400年支组（3 913±1 753）pg/mL与吸烟指数 > 400年支组（4 936±1 752）pg/mL的差异有统计学意义（*P*=0.047）。研究^[9, 10, 11]^报道NSCLC患者吸烟与*EGFR*突变有关，不吸烟的患者应用吉非替尼治疗效果好。本研究结果显示吸烟对EGFR表达量也有影响，*EGFR*突变与表达量之间可能也存在一定的相互作用，值得我们进一步探讨；⑤NSCLC患者手术前后血清EGFR蛋白表达水平显示出明显差异，考虑主要原因为肿瘤负荷减小，肿瘤分泌的EGFR减少。正常人每日约需要摄入（2 000-2 500）mL水，手术前患者每日输液量大约为（500-1 000）mL，手术后患者每日输液量大约为（1 000-1 500）mL，考虑到手术中失水和手术后短暂禁食禁水的影响，术后采集的外周血由于输液被稀释的因素基本可以忽略。而手术后表达水平在不同病理类型差异明显，腺癌组的表达明显较鳞癌组和其它组高；⑥本研究EGFR表达在肺癌组和良性肺病组血清中未显示出统计学差异（*P*=0.932），可能与良性肺病组样本量小有关，但多数报道认为血清EGFR在肺癌患者和正常人之间的表达是有差异的。

综上所述：NSCLC患者EGFR肿瘤组织mRNA和外周血中表达经直线相关分析存在相关性，外周血检测创伤小且方便可行，有可能替代肿瘤组织，具有相似的生物学标记的意义；外周血EGFR蛋白的高表达水平可能作为淋巴结转移的标记物，对肺癌的早期诊断转移或微转移、个体化治疗的开展提供一个更为简单的检测方法。
